# A Steel Wire Stress Measuring Sensor Based on the Static Magnetization by Permanent Magnets

**DOI:** 10.3390/s16101650

**Published:** 2016-10-06

**Authors:** Dongge Deng, Xinjun Wu, Su Zuo

**Affiliations:** School of Mechanical Science & Engineering, Huazhong University of Science and Technology, Wuhan 430074, China; dengdongge@mail.hust.edu.cn (D.D.); u201210902@mail.hust.edu.cn (S.Z.)

**Keywords:** stress evaluation, steel wire, static magnetization, permanent magnet, Hall element arrays

## Abstract

A new stress measuring sensor is proposed to evaluate the axial stress in steel wires. Without using excitation and induction coils, the sensor mainly consists of a static magnetization unit made of permanent magnets and a magnetic field measurement unit containing Hall element arrays. Firstly, the principle is illustrated in detail. Under the excitation of the magnetization unit, a spatially varying magnetized region in the steel wire is utilized as the measurement region. Radial and axial magnetic flux densities at different lift-offs in this region are measured by the measurement unit to calculate the differential permeability curve and magnetization curve. Feature parameters extracted from the curves are used to evaluate the axial stress. Secondly, the special stress sensor for Φ5 and Φ7 steel wires is developed accordingly. At last, the performance of the sensor is tested experimentally. Experimental results show that the sensor can measure the magnetization curve accurately with the error in the range of ±6%. Furthermore, the obtained differential permeability at working points 1200 A/m and 10000 A/m change almost linearly with the stress in steel wires, the goodness of linear fits are all higher than 0.987. Thus, the proposed steel wire stress measuring sensor is feasible.

## 1. Introduction

Because of good mechanical performance, steel cables have been widely used in industrial production and infrastructure construction. As key load-bearing components, their stress state is closely related to the structural stability and safety. Thus, it is of great significance to evaluate the stress in steel cables. The stress in ferromagnetic specimen can be measured by electromagnetic methods, because magnetic properties of ferromagnetic materials change with the applied stress [1]. To obtain the magnetic feature parameter for evaluating the stress, the tested specimen should be magnetized by excitation fields at first. According to whether the excitation field is artificial or not, electromagnetic methods can be divided into the passive magnetic method and the active magnetic method [2,3].

In passive magnetic methods such as the metal magnetic memory (MMM) technique also known as the residual magnetic field (RMF) technique, without application of an excitation field, the tested specimen is in the presence of ambient magnetic fields such as the geomagnetic field. The measured residual magnetic flux density parallel and perpendicular to the surface of the specimen correlate closely with the applied stress [2]. Though artificial excitation field is not needed, the weak detection signal in this technique is easily influenced by the external environment [4,5], it is difficult to measure the stress in steel cables quantitatively by this technique. In active magnetic methods, the steel cable is magnetized by an excitation field at first. Static magnetization, harmonic magnetization and pulse magnetization have been used to evaluate the stress in steel cables. Under the static magnetization, the magnetic field strength parallel to the magnetization direction measured in proximity to the steel cable is used to evaluate the stress [6]. The measurement is conducted at the uniformly magnetized region. Thus, the feature parameter is obtained at a constant working point. It is hard to find the proper working point in this case [7]. Adopting harmonic magnetization, the eddy current technique has also been used to characterization of stress in steel cables [8]. The impedance [9,10] or the induced voltage [11] of an electromagnetic coil is utilized as feature parameters. The research results indicate the close relationship between the feature parameters and the stress in a steel cable with a special size. However, the size effect [12] caused by eddy current in dynamic magnetization may lead to different results for steel cables of different size. The size effect can be reduced when the sample is magnetized to technically saturate. Pulse magnetization is usually adopted to magnetize the sample to the saturation for its low power consumption and small heat production [13]. It is widely used in the Elasto-magnetic (EM) sensor technique [14,15] to evaluate the stress in steel cables.

The EM sensor usually consists of a primary excitation coil and a secondary induction coil. It can directly obtain the incremental permeability to measure the actual stress in a noncontact way [16,17,18]. Thus, it has been used to monitor the stress in steel cables on field more than ten years. The application effect of the EM sensor is good except the installation is complicated [19]. Because it is inevitable to wind both coils on steel cables, a Smart Elasto-Magneto-Electric (EME) Sensor [20,21] which can be installed more easily is recently put up by Duan et al. for stress evaluating. The Magneto-Electric (ME) sensing unit made of a ME-laminated composite is used to take place of the secondary induction coil. Thus, there is no need to wind the second coil on steel cables. However, the primary excitation coil still has to be wound [22]. Therefore, this paper is intended to develop a feasible stress measuring sensor which is easier to install. Specially, as a preliminary study, a new stress measuring sensor is proposed to test Φ5 and Φ7 steel wires frequently used in steel cables. Without using the excitation coil and the induction coil, the proposed sensor obtain the feature parameter related to the stress under the static magnetization by permanent magnets.

This paper is organized as follows. Section 2 interprets the principle of the proposed sensor. In Section 3, the sensor, which applies to both Φ5 and Φ7 steel wires, is developed. Section 4 tests the performance of this sensor by experiments. At last, Section 5 lists the conclusion and an outline of the further work.

## 2. The Principle

**Figure 1:**
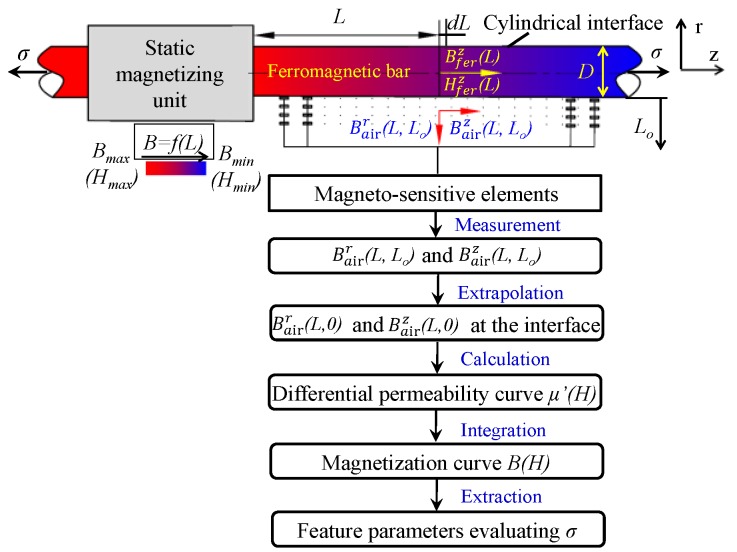
The principle of the proposed steel stress measuring sensor.

Figure 1 shows the principle of the proposed sensor. The sensor is mainly composed of a static magnetizing device and magneto-sensitive elements. The principle is based on the method for measuring the magnetization curve of cylindrical bar specimen under static magnetization [23,24] put up by the author.

As described in Figure 1, under the excitation of the static magnetizing device, a spatial magnetic field *B* = *f*(*L*) varying along the axial position *L* is induced in the cylindrical ferromagnetic bar. Magneto-sensitive elements are used to measure the radial and axial magnetic flux densities $B_{air}^{r}$(*L*, *L_o_*) and $B_{air}^{z}$(*L*, *L_o_*) at different lift-offs *L_o_* in the region where the distribution of axial magnetic flux density $B_{fer}^{z}$(*L*) within the cross section and radial magnetic flux density $B_{air}^{r}$(*L*, 0) around the cylindrical interface is uniform for a given position *L*. Then, radial and axial magnetic flux densities $B_{air}^{r}$(*L*, 0) and $B_{air}^{z}$(*L*, 0) at the interface could be extrapolated from the measured $B_{air}^{r}$(*L*, *L_o_*) and $B_{air}^{z}$(*L*, *L_o_*). In this case, differential permeability curve *μ*’(*H*) can be calculated from the extrapolated $B_{air}^{r}$(*L*, 0) and $B_{air}^{z}$(*L*, 0) according to the continuity of the tangential magnetic field strength, the Gauss’ law for magnetism, Rayleigh relation in Rayleigh region and the law of approach to saturation. Furthermore, magnetization curve *B*(*H*) could be integrated from the calculated *μ*’(*H*). At last, feature parameters at different magnetic field strengths can be extracted to evaluate the stress *σ* in the ferromagnetic bar.

In the above steps to obtain feature parameters evaluating the stress *σ*, it is crucial to obtain magnetization curve *B*(*H*) from the extrapolated $B_{air}^{r}$(*L*, 0) and $B_{air}^{z}$(*L*, 0). The corresponding steps are listed as follows:

(1) The axial magnetic flux density variation *d*[$B_{fer}^{z}$(*L*)] across the unit length *dL* is got from the radial magnetic flux density $B_{air}^{r}$(*L*, 0) at the interface as Equation (1) according to the Gauss’ law for magnetism.

(1) $$d\left[B_{fer}^{z}(L)\right]=\frac{4B_{air}^{r}(L,0)dL}{D}$$

(2) The axial magnetic field strength variation *d*[$H_{fer}^{z}$(*L*)] across the unit length *dL* is got from the axial magnetic flux density $B_{air}^{z}$(*L*, 0) at the interface as Equation (2) according to the continuity of the tangential magnetic field strength.

(2) $$d\left[H_{fer}^{z}(L)\right]=\left[B_{air}^{z}(L+dL,0)-B_{air}^{z}(L,0)\right]/\mu_{0}$$

(3) The differential permeability $\mu_{in}^{\prime }$(*L*) can be got from Equation (3) which is obtained by dividing Equation (2) with Equation (1). Then combining with the axial magnetic field strength $H_{fer}^{z}$(*L*) at different axial position *L*, the differential permeability $\mu_{in}^{\prime }$($H_{fer}^{z}$) _(*Hmin* ≤ $H_{fer}^{z}$ ≤ *Hmax*)_ at the measurement region can be got.

(3) $$\mu_{in}^{\prime }(L)=\frac{d\left[B_{fer}^{z}(L)\right]}{d\left[H_{fer}^{z}(L)\right]}=\frac{4B_{air}^{r}(L,0)dL}{D\left[H_{air}^{z}{(L+dL,0)-\;H}_{air}^{z}(L,0)\right]}$$

(4) According to Rayleigh relation in Rayleigh region [25] and the law of approach to saturation [26] described in Equations (4) and (5), respectively, the differential permeability $\mu_{in}^{\prime }$($H_{fer}^{z}$) _(0 ≤ $H_{fer}^{z}$ ≤ *Hsa*)_ at the magnetic field strength $H_{fer}^{z}$ ∈ [0, *H_sa_*] can be extrapolated from the $\mu_{in}^{\prime }$($H_{fer}^{z}$) _(*Hmin* ≤ $H_{fer}^{z}$ ≤ *Hmax*)._ Where, *H_sa_* denotes the magnetic field strength which can magnetize the specimen to technical saturation.

(4) $$\mu_{in}^{\prime }=dB/dH=\mu_{0}\mu_{r}+2\nu H$$

(5) $$\mu_{in}^{\prime }=\frac{dB}{dH}=\mu_{0}\left[M_{s}(\frac{a}{H^{2}}+\frac{2b}{H^{3}}+\frac{3c}{H^{4}}+\cdots )+1+\chi_{p}\right]$$

In Equation (4), the parameter *υ* is called Rayleigh constant. 2*υ* is equal to ${d\mu }_{in}^{\prime }$/*dH*. In Equation (5), *μ*_0_ is the permeability of free space, *M_s_* is the saturation magnetization, and the parameter “*a*” is called magnetic hardness [27]. Parameter *b*, *c* and the other higher parameters without any particular definition are constants related to the magnetocrystalline anisotropy of the material. The parameter *χ_p_* is the paramagnetic susceptibility.

Equation (5) is a general formula to describe the relationship between the differential permeability $\mu_{in}^{\prime }$ and the applied magnetic field *H*. This equation includes too many terms that it cannot be used conveniently in practice. Thus, this equation needs to be simplified in some cases. In the most important special case, the terms about *b*, *c* and the other higher terms are neglected. This leads to WEISS saturation law, which can be described in Equation (6) [27].

(6) $$\mu_{in}^{\prime }=\frac{dB}{dH}=\mu_{0}\left[M_{s}(\frac{a}{H^{2}})+1\right]$$

The Equation (6) has been verified to be able to model the magnetization of prestressing steel without applied stress near saturation accurately [27]. When taking the stress dependency into account, the Equation (6) should be modified to Equation (7) as follows.

(7) $$\mu_{in}^{\prime }=\frac{dB}{dH}=\mu_{0}\left[M_{s}(\frac{a}{H^{2}})(1+\frac{\sigma }{c_{\sigma }})+1\right]$$

where *c_σ_* is called as magnetoelastic stress parameter. Compared with Equation (6), the terms related to the stress *σ* have been added into Equation (7). As can be seen from Equation (7), the differential permeability $\mu_{in}^{\prime }$ in the region approach to saturation changes linearly with the applied stress *σ* when the magnetic field strength is constant.

(5) At last, the Magnetization curve *B*(*H*) can be found by the point-by-point integration of the differential permeability $\mu_{in}^{\prime }$($H_{fer}^{z}$) at the interval [0, *H_sa_*] described in Equation (8). The *B*(*H*) in Equation (8) is a variable limit integral function. Where *H* is the function variable and $H_{fer}^{z}$ is the integration variable.

(8) $$B\left(H\right)=\int_{0}^{H}\frac{dB_{fer}^{z}}{dH_{fer}^{z}}dH_{fer}^{z}=\int_{0}^{H}\mu_{in}^{\prime }\left(H_{fer}^{z}\right)dH_{fer}^{z},\;H\in \left[0,H_{sa}\right]$$

Therefore, with the help of an magnetizing unit providing appropriate static magnetization, the magnetization curve *B*(*H*) of a cylindrical bar specimen could be obtained to evaluate the stress. Based on this, a sensor for evaluating the stress in Φ5 and Φ7 steel wires is developed in this paper.

## 3. The Development of the Proposed Sensor

According to the principle illustrated above, the sensor should consist of a proper static magnetization unit and an appropriate magnetic field measurement unit containing magneto-sensitive elements.

### 3.1. Static Magnetization Unit

**Figure 2:**
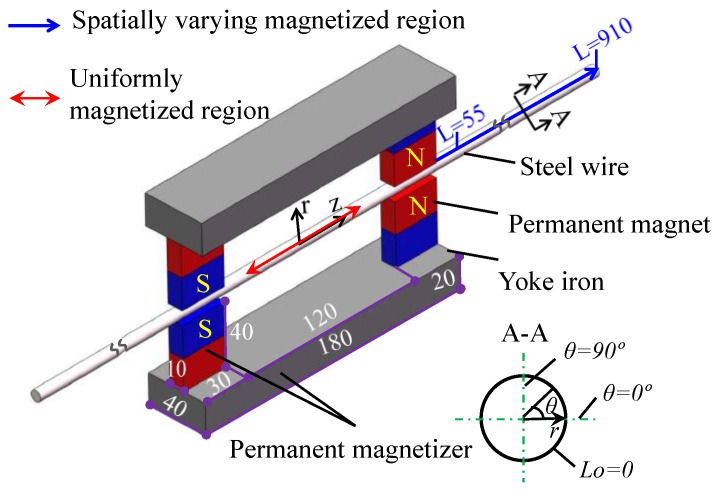
The dimensions of static magnetization unit for evaluating the stress in steel wires (Unit: mm).

The static magnetization unit should satisfy the uniform distribution conditions for the magnetic flux density. Namely, under the excitation of the magnetization unit, the distribution of axial magnetic flux density $B_{fer}^{z}$(*L*) within the cross section and radial magnetic flux density $B_{air}^{r}$(*L*, 0) around the cylindrical interface are uniform for a given position *L*. In principle, DC coils [23] or circular permanent magnets, which can be placed coaxial with the steel wire, should be adopted as the static magnetization unit. In such cases, the uniform distribution of radial magnetic flux density $B_{air}^{r}$(*L*, 0) around the cylindrical interface can be easily satisfied. However, DC coils is hard to be wound on the steel wire. The circular permanent magnets could be only installed from the end of the steel wire, which is impossible when the steel wire is anchored. Thus, a static magnetization unit shown in Figure 2 was adopted by the author to magnetize the 2 m length Φ7 steel wire [24].

**Figure 3:**
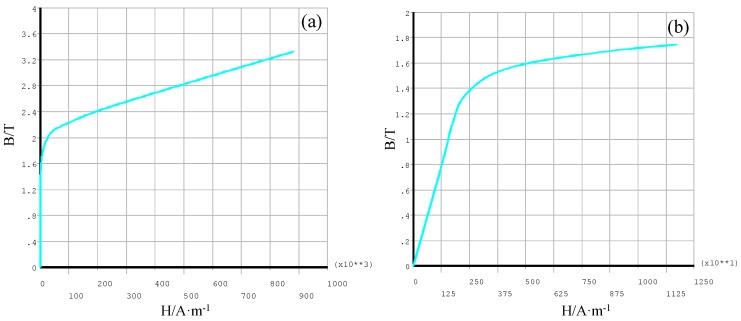
The B-H curves for the yoke iron (**a**) and the steel wire (**b**).

The static magnetization unit consists of two permanent magnetizers which are symmetrically arranged on the center of the steel wire. The permanent magnetizer is composed of the yoke iron with a high level of permeability and NdFeB N35 magnets. Different form the present electromagnetic method adopting static magnetization [6] where the uniformly magnetized region is served as the testing region. The spatially varying magnetized region under the excitation of the static magnetization unit is utilized as the testing region. The field varying along the axial position *L* in this region is adopted as the excitation field. This is because that only using this excitation field can magnetic parameters at different magnetic field strengths be obtained under a static magnetization. In addition, a similar field under a static magnetization has been adopted by other researchers to obtain the magnetic hysteresis loss of the inspected members [28]. In the region from *L* = 55 mm to *L* = 910 mm, the uniform distribution conditions for the magnetic flux density is satisfied [24]. Furthermore, there is no size effect caused by eddy current. This static magnetization unit may also be suitable for Φ5 steel wire. Thus, a 3D finite element model for magnetizing Φ5 steel wire with this static magnetization unit is created by ANSYS software to analyze the distribution of the magnetic flux density. The geometrical size of the model is shown in Figure 2. The lift-off of the magnets from the steel wire is 1 mm. The air surrounding the static magnetization unit and the steel wire is a 3-m length cylinder with the radius of 200 mm. The 3-D magnetic scalar solid 96 element is used to mesh the established entity. The entities in the model are associated with the corresponding material properties, where the coercivity, remanent induction and relative permeability of the magnets are set to 876400 A/m, 1.184 T and 1.075, respectively according to the performance testing report provided by the supplier (Shenzhen Xunci Magnetism Co., Ltd., China). The B-H curves for the yoke iron and the steel wire are shown in Figure 3. The B-H curve for the steel wire is measured by magnetizing coils and measuring coils in our laboratory, the corresponding measurement system has been given in an earlier paper [29].

**Figure 4:**
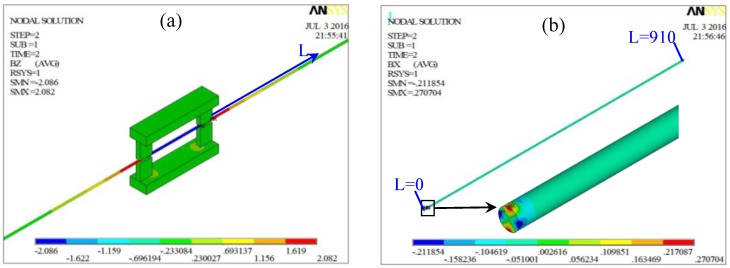
The calculated axial magnetic flux density (**a**) and the radial magnetic flux density (**b**).

After establishing the model, the difference scalar potential (DSP) method is adopted and the Jacobi Conjugate Gradient (JCG) iterative equation solver is used to solve the model quickly and accurately. The calculated axial magnetic flux density and the radial magnetic flux density in this model are shown in Figure 4a,b, respectively.

**Figure 5:**
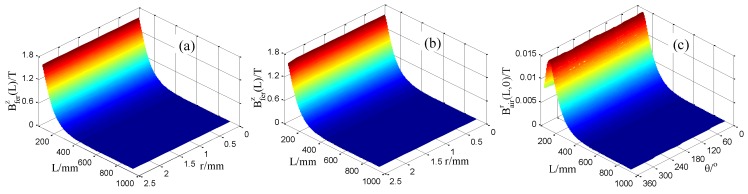
The distribution of $B_{fer}^{z}$(*L*) in the plane *θ* = 0°(**a**), $B_{fer}^{z}$(*L*) in the plane *θ* = 90°(**b**) and $B_{air}^{r}$(*L*,0) in the cylindrical interface *r* = 2.5 mm (**c**) from *L* = 55 mm to *L* = 910 mm.

The magnetic flux densities in the steel wire is the vector addition of the magnetic flux density created by the two permanent magnetizers according to the magnetic field superposition principle which is frequently to be used to create a special magnetic field such as the homogeneous magnetic field created by the Helmholtz coil [30]. As depicted in Figure 4a, the axial magnetic flux density is varying along the axial location *L*. While the distribution of the axial magnetic flux density in the cross section of the spatially varying magnetized region seems uniform. Figure 4b describes the radial magnetic flux density in the steel wire on the right side of the magnetization unit. In the section near the axial location *L* = 0 namely the right end of the magnetization unit, the distribution of the radial magnetic flux density around the cylindrical interface is not homogeneous. The bad homogeneity in the region near the magnetizers is due to that the two permanent magnetizers are not rotational symmetry about the axial line of the steel wire. In such case, when the axial position *L* is near the right end of the magnetization unit, the distribution of the superimposed magnetic field is complex through the vector addition of the magnetic field. However, when the axial position *L* has some distance from the magnetization unit, a small length of the steel wire can be regarded as a particle approximately and the homogeneity of the magnetic field becomes better, which can be indicated in Figure 4. Thus, the magnetic flux density in the region from *L* = 55 mm to *L* = 910 mm is extracted to further analyze the distribution uniformity. The distribution of the axial magnetic flux density $B_{fer}^{z}$(*L*) in the plane *θ* = 0°, the axial magnetic flux density $B_{fer}^{z}$(*L*) in the plane *θ* = 90° and the radial magnetic flux density $B_{air}^{r}$(*L*, 0) in the cylindrical interface *r* = 2.5 mm are shown in Figure 5a–c.

Figure 5 depicts that in the region from *L* = 55 mm to 910 mm, at a fixed axial location “*L*”, $B_{fer}^{z}$(*L*) in the planes *θ* = 0° and *θ* = 90° are approximately identical, $B_{air}^{r}$(*L*, 0) around the cylindrical interface *r* = 2.5 mm are also approximately identical. Namely, in this region, the distribution of the axial magnetic flux density in the cross section of the steel wire and the radial magnetic flux density in the cylindrical interface can be both considered uniform for a given position *L*. Thus, under the excitation of the static magnetization unit shown in Figure 2, the uniform distribution conditions for the magnetic flux density in Φ5 steel wire can be satisfied. That is to say, not only appropriate for Φ7 steel wire, this static magnetization unit can also be used to magnetize Φ5 steel wire.

**Figure 6:**
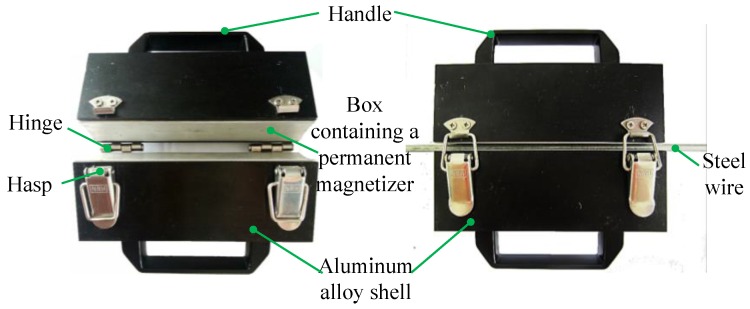
The developed static magnetization unit.

Based on the analysis above, the static magnetization unit is developed, as shown in Figure 6. An aluminum alloy box is used to contain the permanent magnetizer described in Figure 2. Then, an aluminum alloy shell combining the handle is used to package the box. At last, the two aluminum alloy shells are connected by Hinges and Hasps to constitute the magnetization unit. Unlocking the hasp, the magnetization unit is open to fold the tested steel wire. Then locking the hasp, the magnetization unit is closed to magnetize the steel wire.

### 3.2. Magnetic Field Measurement Unit

According to the principle illustrated in Section 2, the magnetic field measurement unit should contain the magneto-sensitive elements which can pick up the radial and axial magnetic flux densities $B_{air}^{r}$(*L*, *L_o_*) and $B_{air}^{z}$(*L*, *L_o_*) at different lift-offs *L_o_* from the steel wire under the static magnetization. To meet this requirement, the magneto-sensitive elements should apply to static magnetic field measurement, have high spatial resolution, large linear measuring range and high sensitivity. In addition, the elements should be easily configured in array.

Induction coils, GMR and Hall elements are frequently-used in magnetic field measurement. Induction coils are not sensitive to the static magnetic field. GMR has high sensitivity but a low linear measuring range. While the Linear Hall elements have many advantages: ratiometric linear output proportional to the magnetic field, high spatial resolution for its small size, immune to the packing stress and the temperature variation for its on-chip temperature compensation and magnetic characteristics robust against mechanical stress. Thus, linear HAL1823 elements in TO92UA Package produced by MICRONAS are adopted in this work. Its volume and sensitive area are 4 × 3 × 1.5 mm^3^ and 0.2 × 0.1 mm^2^, respectively. Its sensitivity and linearity measuring range are 2.5 mv/G and ±1000 G, respectively, which can satisfy the request for magnetic field measurement in this work.

**Figure 7:**
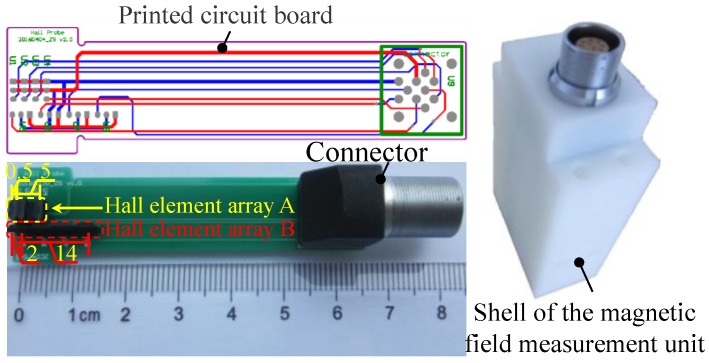
The magnetic field measurement unit for evaluating the stress in steel wires.

To easily configure hall elements in array, a printed circuit board (PCB) is designed and developed, as shown in Figure 7. Then the Hall element array “A” containing 4 Hall elements for measuring the radial magnetic flux densities, the Hall element array “B” containing 4 Hall elements for measuring the axial magnetic flux densities and the connector are soldered to the PCB.

Under the configuration depicted in Figure 7, the sensitive area of Hall element array “A” and “B” can be easily ensured perpendicular to the radial and axial direction. Furthermore, the position of Hall elements can be easily fixed. They are placed together and closely to the end of the PCB which is contacted to the surface of the steel wire. Thus, the minimal and maximal lift-offs of Hall elements of array “A” are 0.5 mm and 5 mm, respectively. The space between the adjacent Hall elements of array “A” is 1.5 mm. The minimal and maximal lift-off of Hall elements of array “B” are 2 mm and 14 mm, respectively. The space between the adjacent Hall elements of array “B” is 4 mm. They can measure radial magnetic flux densities at the lift-off *L_o_* = 0.5, 2, 3.5, 5 mm and axial magnetic flux densities at the lift-off *L_o_* = 2, 6, 10, 14 mm. Then, to fix and protect the PCB, a shell is made, as shown in Figure 7. The shell is made from the resin which is non-ferromagnetic by 3D printing. This material won’t affect the measurement result of the Hall elements.

### 3.3. The Assembly of the Proposed Sensor

**Figure 8:**
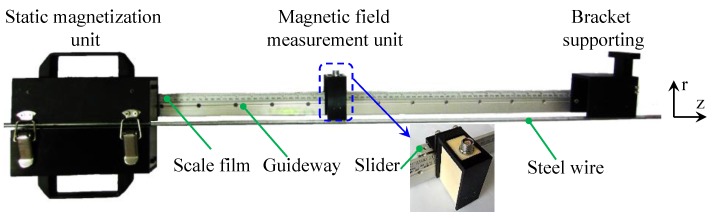
The assembly of the proposed sensor.

Figure 8 shows the assembled sensor, which consists of the static magnetization unit, the magnetic field measurement unit, the bracket supporting and the guideway.

The static magnetization unit and the bracket supporting are connected with the different ends of the guideway. The contact surfaces of the static magnetization unit and the bracket supporting with the steel wire are coplanar to make the guideway parallel to the steel wire. The magnetic field measurement unit is connected with the slider which can scan along the guideway. Thus, the magnetic field measurement unit can pick up the axial and radial magnetic flux density at different lift-offs in the spatially varying magnetized region of the steel wire. When measuring the stress in steel wires, the static magnetization unit is open to fold the steel wire. Then closing the static magnetization unit, the sensor can be installed on the steel wire. The installation is convenient for no need to wind coils.

## 4. Experimental Investigation of the Proposed Sensor

To investigate the performance of the proposed sensor, steel wires specimen are prepared and the experimental setup is built. In the first place, the measurement accuracy of the sensor for magnetization curve is studied, which is the first step to obtain the steel wire stress. Secondly, the feasibility to evaluate stresses in the Φ5 steel wire by the sensor is analyzed. At last, the performance of the sensor for the Φ7 steel wire is tested.

### 4.1. Specimen Preparation

Three 2 m length Φ5 steel wires and three 2 m length Φ7 steel wires are adopted as the specimens. The steel wires are provided by LIUZHOU OVM ENGINEERING CO., LTD. They are made from the SWRS82B steel whose chemical composition is shown in Table 1.

Through the continuous drawing, hot-dip galvanizing and stabilizing treatment, their tensile strength can reach 1670 MPa. Because of such good mechanical properties, they are usually used to produce parallel wire cables. The axial stress in them is about 600 MPa in the practical application.

**Figure 9:**
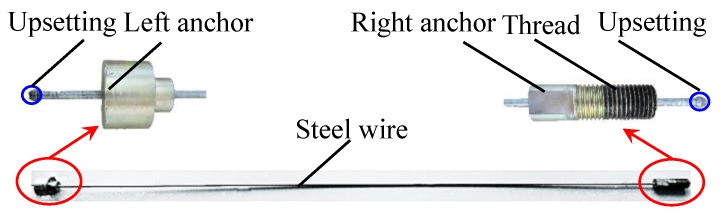
The tested steel wires specimen.

The tested steel wires are shown in Figure 9, the upsetting process is conducted on both ends of the steel wire. The left and right anchors are adopted to ease of anchoring the steel wire. Furthermore, the thread is machined on the right anchor to ease of applying stress in the steel wire.

### 4.2. The Experimental Setup

**Figure 10:**
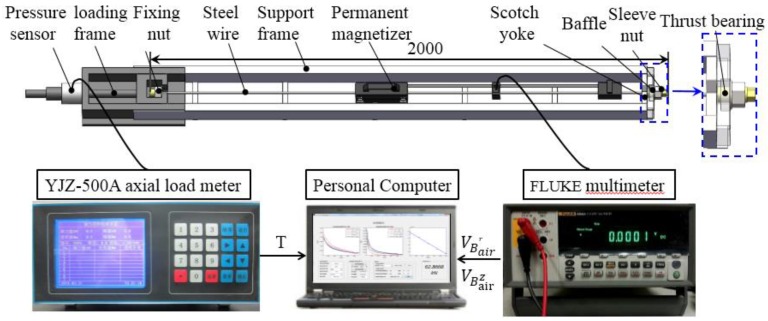
The experimental system to test the performance of the sensor.

Figure 10 shows the experimental system to test the performance of the proposed sensor. A 2 m length steel wire is anchored on the loading machine. The loading machine mainly consists of the loading frame, fixing nut, support frame, scotch yoke, baffle and the sleeve nut. Screwing the sleeve nut on the right, the steel wire can be loaded. During the loading, the scotch yoke prevents the steel wire from rotating. The scotch yoke, support frame and the baffle are all made of the non-ferromagnetic aluminum alloy material. This material will not affect the magnetization of the steel wire. The sensor is installed on the steel wire. The center of its static magnetization unit is 1000 mm from the right end of the steel wire.

When conducting the experiments, the applied load *T* is acquired by the pressure sensor connected with an axial load meter (YJZ-500A, Jinan at Industry and trade co., LTD, Jinan, Shandong, China) and transmitted to the personal computer. The steel wire is magnetized by the static magnetization unit. The region from *L* = 55 mm to 345 mm is adopted as the magnetic measurement region. As the axial magnetic flux density $B_{fer}^{z}$ at the location *L* = 345 mm is already close to zero, which can be seen in Figure 5. The distance between two adjacent measuring points is 10 mm. The radial and axial magnetic fluxes at different lift-offs in this region are measured by the magnetic field measurement unit connected with a FLUKE8808A multimeter. Specially, Hall element arrays in the measurement unit converts the magnetic signals into the voltage signals. The FLUKE8808A multimeter picks up the voltage signals ${VB}_{\mathrm{air}}^{r}$ and ${VB}_{\mathrm{air}}^{z}$ and uploads them to the personal computer. Combining the load *T* with voltage signals ${VB}_{\mathrm{air}}^{r}$ and ${VB}_{\mathrm{air}}^{z}$, the differential permeability curve and magnetization curve of the steel wire are calculated by the software Matlab R2013a. Further, the feature parameter to evaluate axial stress in the steel wire is extracted.

By using the experimental system described in Figure 10, the performance of the proposed sensor is tested through several experiments.

### 4.3. Measurement Accuracy of the Sensor for Magnetization Curve

**Figure 11:**
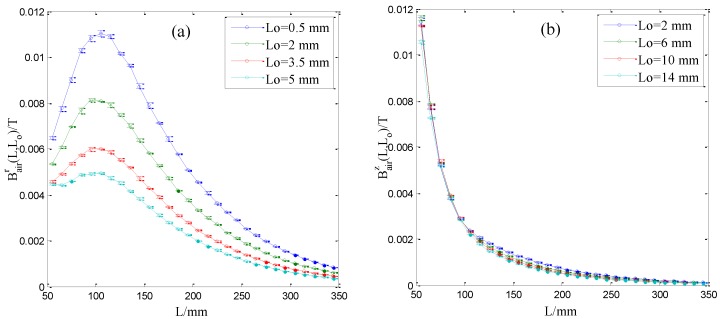
Three repeated measurements of radial (**a**) and axial (**b**) magnetic flux densities at different lift-offs in the measurement region.

Firstly, the measurement accuracy of the sensor for magnetization curve is investigated, which is the basis of the stress evaluation. The proposed sensor is put on a Φ5 steel wire whose axial stress is zero. Three repeated measurements of radial and axial magnetic flux densities at different lift-offs are conducted. The measured data are shown in Figure 11.

**Figure 12:**
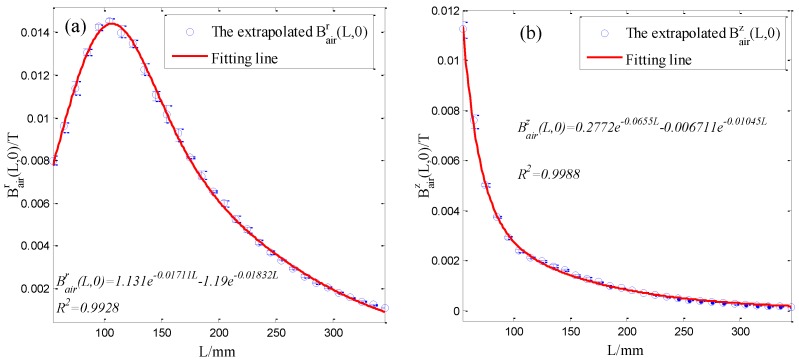
The extrapolated radial (**a**) and axial (**b**) magnetic flux densities at the interface and the corresponding fitting lines.

Figure 11 demonstrates the average, minimum and maximum values of the measured radial and axial magnetic flux densities. It can be seen that the measurement repeatability is good. Then, according to the principle in Section 2, radial and axial magnetic flux densities $B_{air}^{r}$(*L*, 0) and $B_{air}^{z}$(*L*, 0) at the interface are extrapolated from the measured $B_{air}^{r}$(*L*, 0) and $B_{air}^{z}$(*L*, 0) to obtain the magnetization curve. The extrapolation method is referenced to previous research [31,32,33]. Specially, at a fixed measuring point, a third-order polynomial is used to fit the relationship between the radial magnetic flux density $B_{air}^{r}$*(L*, *L_o_)* and the lift-off *L_o_* shown in Figure 11a. Then the fitting coefficients can be obtained. The polynomial value at *L_o_* = 0 is considered as the radial magnetic flux densities $B_{air}^{r}$(*L*, 0) at the interface. Similarly, the radial magnetic flux densities at other measuring points can be obtained. The extrapolation method for the axial magnetic flux densities $B_{air}^{z}$(*L*, 0) is the same with $B_{air}^{r}$(*L*, 0). The extrapolated radial and axial magnetic flux densities at the interface are shown in Figure 12.

**Figure 13:**
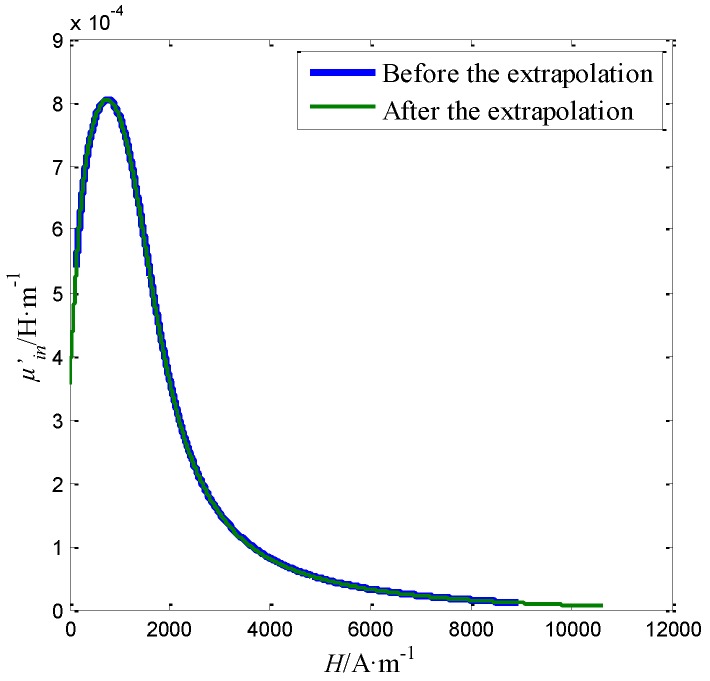
The differential permeability curve before and after the extrapolation.

In order to calculate the differential permeability curve, the extrapolated $B_{air}^{r}$(*L*, 0) and $B_{air}^{z}$(*L*, 0) should be fitted to obtain the corresponding distribution functions. The double exponential function is used to fit extrapolated $B_{air}^{r}$(*L*, 0) and $B_{air}^{z}$(*L*, 0) by Matlab R2013a. The obtained distribution functions for $B_{air}^{r}$(*L*, 0) and $B_{air}^{z}$(*L*, 0) are shown in Figure 12a,b, respectively. The goodness of the fits for $B_{air}^{r}$(*L*, 0) and $B_{air}^{z}$(*L*, 0) are 0.9928 and 0.9988, respectively. The fitting results are good. Then, taking *dL* = 0.01 mm, according to Equations (1)–(3) described in Section 2, the differential permeability curve $\mu_{in}^{\prime }$ at the magnetic field strength $H_{fer}^{z}$∈ [141.5, 8944.7] applied to the measurement region is calculated from the average $B_{air}^{r}$(*L*, 0) and $B_{air}^{z}$(*L*, 0), which is shown as the blue line in Figure 13.

The differential permeability $\mu_{in}^{\prime }$($H_{fer}^{z}$) _(0 ≤ $H_{fer}^{z}$ ≤ *Hsa*)_ is further extrapolated from $\mu_{in}^{\prime }$($H_{fer}^{z}$) according to the Rayleigh relation in Rayleigh region described in Equation (4) and the law of approach to saturation described in Equation (7) in Section 2. In such cases, the differential permeability $\mu_{in}^{\prime }$ in the Rayleigh region and the region approach to saturation can be both obtained by the linear extrapolation. The extrapolated points for both regions are listed in Table 2.

**Figure 14:**
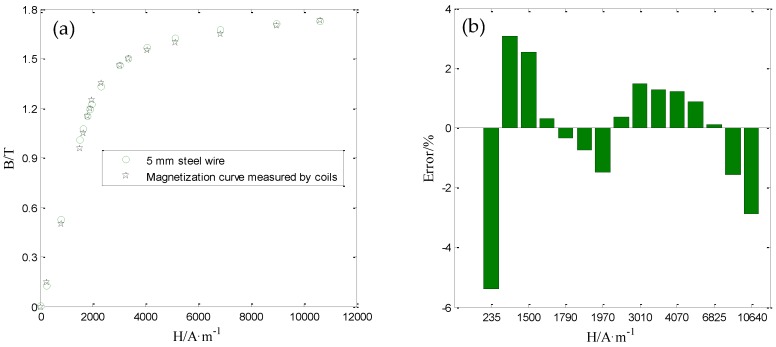
The calculated magnetization curve (**a**) and the corresponding error (**b**).

The obtained differential permeability $\mu_{in}^{\prime }$($H_{fer}^{z}$) _(0 ≤ $H_{fer}^{z}$ ≤ *Hsa*)_ is shown as the green line in Figure 13. At last, the magnetization curve for the steel wire can be obtained by the integration shown in Equation (8). The magnetization curve measured by the proposed sensor is shown in Figure 14a. Comparing with the magnetization curve measured by coils shown in Figure 3b, the error of the magnetization curve measured by the proposed sensor can be obtained and shown in Figure 14b.

As shown in Figure 14b, the error under different magnetic field strengths is different. All the errors are within the range of ±6%. Namely, the proposed sensor can obtain the magnetization curve for the steel wire accurately. Based on this, the proposed senor is used to evaluate the axial stress in steel wires.

### 4.4. The Performance of the Sensor for Steel Wires with 5 mm in Diameter

**Figure 15:**
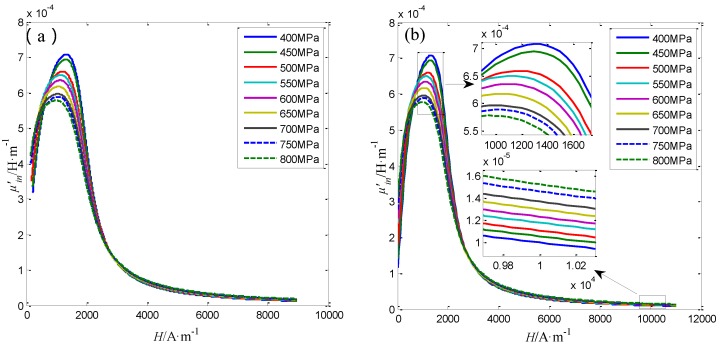
The differential permeability curves before (**a**) and after (**b**) the extrapolation for No.1 Φ5 steel wire under different axial stresses.

All the three Φ5 steel wires are used in the stress evaluating experiment, the axial stress applied to the Φ5 steel wire is changed from 400 MPa to 800 MPa with the step of 50 MPa. Under the applied axial stresses, the radial and axial magnetic flux densities $B_{air}^{r}$(*L*, *L_o_*) and $B_{air}^{z}$(*L*, *L_o_*) at different lift-offs *L_o_* from the Φ5 steel wire are measured by the proposed sensor. Three repeated measurements are also conducted for each Φ5 steel wire. The magnetic property of the steel wire are calculated form the measured $B_{air}^{r}$(*L*, *L_o_*) and $B_{air}^{z}$(*L*, *L_o_*) according to the method described in Section 4.3. Specially, the obtained average differential permeability curves before and after the extrapolation for the No.1 Φ5 steel wire under different axial stresses are shown in Figure 15.

**Figure 16:**
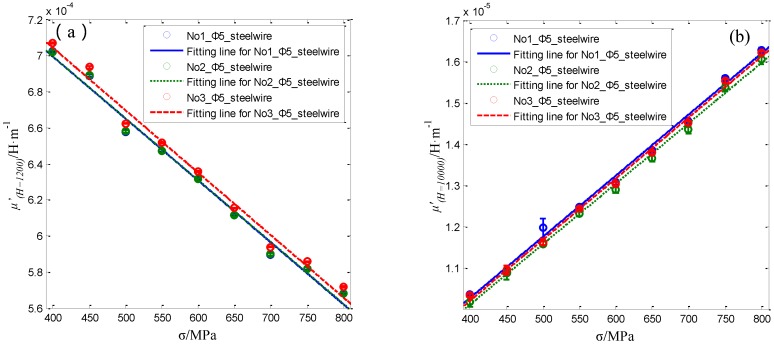
The relationship of axial stress in the three Φ5 steel wires with differential permeability at working points *H* = 1200 A/m (**a**) and *H* = 10,000 A/m (**b**).

The differential permeability curves under various axial stress are different from each other. As can be seen in Figure 15b, the differential permeability *μ*’*_(H = 1200)_* at the working point about 1200 A/m decreases with the stress. While the differential permeability *μ*’*_(H = 10000)_* at the working point about 10,000 A/m increases with the stress. The variation of the differential permeability with the stress is similar with the research results in the reference [34]. The relationship of axial stress in No.1 Φ5 steel wire with differential permeability at *H* = 1200 A/m and *H* = 10,000 A/m are shown as the blue line in Figure 16a,b, respectively.

The blue lines in Figure 16 demonstrates the average, minimum and maximum values of the calculated *μ*’*_(H = 12_*_00*)*_ and *μ*’*_(H = 1_*_0000*)*_ for the No.1 steel wire under different axial stresses. In addition, the average, minimum and maximum values of the *μ*’*_(H = 12_*_00*)*_ and *μ*’*_(H = 1_*_0000*)*_ for the No.2 and No.3 steel wires are calculated and shown as the green and red lines in Figure 16. The average values of both *μ*’*_(H = 12_*_00*)*_ and *μ*’*_(H = 1_*_0000*)*_ for the three Φ5 steel wires are approximately coincident. Furthermore, both of them change almost linearly with the applied stress. The first order polynomial is used to fit the relationship of the average *μ*’*_(H = 12_*_00*)*_ and *μ*’*_(H = 1_*_0000*)*_ with the applied axial stress for the three steel wires. The obtained fitting function and goodness R-square of fitted curves *μ*’*_(H = 12_*_00*)*_ (σ) and *μ*’*_(H = 1_*_0000*)*_ (σ) for the three different Φ5 steel wires are list in Table 3.

As can be seen in Table 3, the R-square of fitted curves *μ*’*_(H = 12_*_00*)*_ (σ) and *μ*’*_(H = 1_*_0000*)*_ (σ) for the three different Φ5 steel wires are all higher than 0.989. This proposed sensor can be used to obtain the proper magnetic feature parameter to evaluate the axial stress in Φ5 steel wire. Comparatively speaking, the R-square of fitted curves *μ*’*_(H = 1_*_0000*)*_ (σ) is better than the R-square of fitted curves *μ*’*_(H = 12_*_00*)*_ (σ) for each Φ5 steel wire.

### 4.5. The Performance of the Sensor for Steel Wires with 7 mm in Diameter

**Figure 17:**
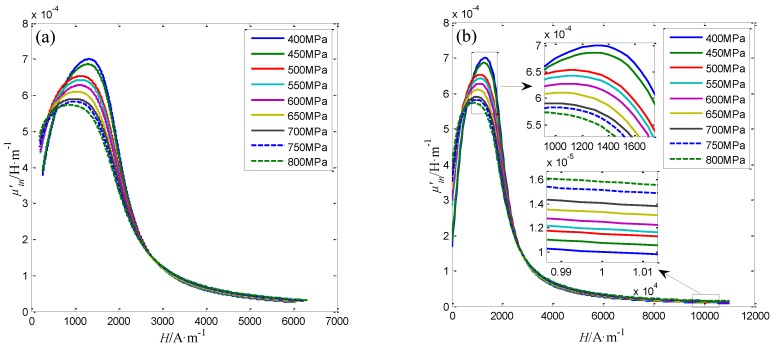
The differential permeability curves before (**a**) and after (**b**) the extrapolation for No.1 Φ7 steel wire under different axial stresses.

At last, the applicability of the sensor for steel wires with 7mm in diameter is studied. Three Φ7 steel wires are used as the specimens. The axial stress applied to Φ7 steel wires is also changed from 400 MPa to 800 MPa with the step of 50 MPa. The measurement steps for the radial and axial magnetic flux densities $B_{air}^{r}$(*L*, *L_o_*) and $B_{air}^{z}$(*L*, *L_o_*) at different lift-offs *L_o_* from the Φ7 steel wire are same with the Φ5 steel wire. In addition, three repeated measurements are conducted for each Φ7 steel wire. The calculation method for the magnetic parameters of the Φ7 steel wire is similar with the Φ5 steel wire. The calculated differential permeability curves before and after the extrapolation for No.1 Φ7 steel wire under different axial stresses are shown in Figure 17.

**Figure 18:**
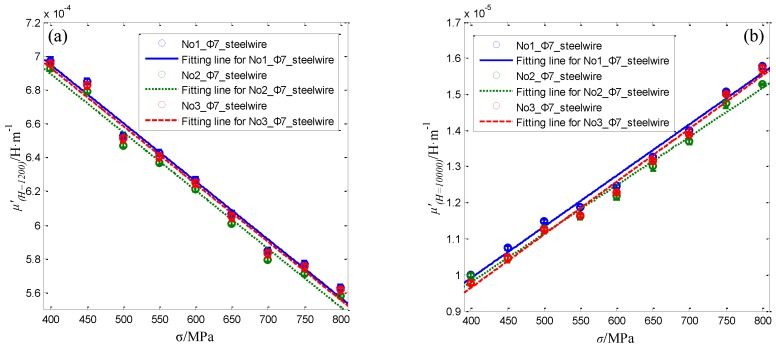
The relationship with the axial stress in the three Φ7 steel wire of differential permeability at working points *H* = 1200 A/m (**a**) and *H* = 10,000 A/m (**b**).

Comparing with Figure 15a, it can be found that under the excitation of the static magnetization unit from the proposed sensor, the maximum magnetic field strength applied to No.1 Φ7 steel wire in the measurement region is smaller than the Φ5 steel wire. However, as seen in the partial enlarged views described in Figure 17b, the change of the differential permeability *μ*’*_(H = 12_*_00*)*_ and *μ*’*_(H = 1_*_0000*)*_ with the axial stress in the Φ7 steel wire is the same with the Φ5 steel wire. Further more, the average, minimum and maximum values of the *μ*’*_(H = 12_*_00*)*_ and *μ*’*_(H = 1_*_0000*)*_ for the three Φ7 steel wires under different axial stresses are calculated and shown in Figure 18.

Comparing with Figure 16, it can be found that the average values of the *μ*’*_(H = 12_*_00*)*_ and *μ*’*_(H = 1_*_0000*)*_ for Φ7 steel wires are slightly different from Φ5 steel wires. However, both the *μ*’*_(H = 12_*_00*)*_ and *μ*’*_(H = 1_*_0000*)*_ for Φ7 steel wires are basically in coincidence and change almost linearly with the applied stress. The fitting function and goodness R-square of first order polynomial fitted curves *μ*’*_(H = 12_*_00*)*_ (σ) and *μ*’*_(H = 1_*_0000*)*_ (σ) for the three different Φ7 steel wires are list in Table 4.

Comparing with Table 3, it can be found that the R-square of fitted curves *μ*’*_(H = 12_*_00*)*_ (σ) and *μ*’*_(H = 1_*_0000*)*_ (σ) for Φ7 steel wires are close to Φ5 steel wires. All the R-squares are higher than 0.987. Thus, this proposed sensor also applies to the stress evaluating in the Φ7 steel wire. Comparatively speaking, the R-square of fitted curves *μ*’*_(H = 1_*_0000*)*_ (σ) is also better than the R-square of fitted curves *μ*’*_(H = 12_*_00*)*_ (σ) for each Φ7 steel wire. This indicates that *μ*’*_(H = 1_*_0000*)*_ may be a more suitable feature parameter for stress evaluating in steel wires by this proposed sensor.

## 5. Conclusions

This paper proposes a stress measuring sensor for steel wires based on the static magnetization by permanent magnets. Comparing to the existing Elasto-magnetic sensor for measuring the stress, primary excitation coils and induction coils are replaced by a static magnetization unit and a magnetic field measurement unit containing Hall element arrays, respectively. Especially, the static magnetization unit is made of permanent magnets and yokes. It can be opened conveniently to fold the steel wire, which can ease the installation of the proposed sensor. Under the excitation of the static magnetization unit, a spatially varying magnetized region in the steel wire is adopted as the measurement region. In this region, the distribution of the axial magnetic flux density in the cross section and the radial magnetic flux density around the cylindrical surface of the steel wire are uniform for a given position *L*. Radial and axial magnetic flux densities at different lift-offs from the steel wire in this region are measured by the measurement unit. Then, the differential permeability curve and the magnetization curve are calculated from the measured magnetic flux density. At last, the differential permeability *μ*’*_(H = 12_*_00*)*_ and *μ*’*_(H = 1_*_0000*)*_ are extracted to evaluate the stress in steel wires.

An experimental system is set up to test the performance of the proposed sensor. Experimental results show that the sensor can measure the magnetization curve of the steel wire accurately with the error located in the range of ±6%. The obtained differential permeability *μ*’*_(H = 12_*_00*)*_ decreases almost linearly with the stress in steel wires. While, the differential permeability *μ*’*_(H = 1_*_0000*)*_ increases almost linearly with the stress in steel wires. The goodness of the corresponding linear fits are higher than 0.987. Thus, the proposed sensor is feasible to evaluate the stress in steel wires.

However, the proposed sensor only applies to evaluating the axial stress in Φ5 and Φ7 steel wires in the laboratory. The sensor still has to be developed to evaluate the stress in other steel components. For example, the influence of the leakage magnetic field between the steel wires needs to be analyzed, when evaluating the stress in steel strands by the sensor. In addition, the influence of plastic protective covers on the signals of the sensor need to be researched, when the sensor is used to evaluate the stress in steel cables on site. These will be the topic in our following research.

## Figures and Tables

**Table 1:** Chemical composition of the SWRS82B steel (wt%).

C	Si	Mn	P	S	Ni	Cr	Cu
0.81	0.22	0.82	0.013	0.014	0.01	0.26	0.01

**Table 2:** The extrapolated points for Rayleigh region and the region approach to saturation.

Working Region	Rayleigh Region	Region Approach to Saturation
(*H_1_*, *μ*’*_in1_*)/(A/m, × 10^−4^ H/m))	(141.5, 5.404)	(8906.9, 0.1088)
(*H_2_*, *μ*’*_in2_*)/(A/m, × 10^−4^ H/m))	(143.0, 5.424)	(8944.7, 0.1073)

**Table 3:** The fitting function and goodness R-square of fitted curves *μ*’*_(H = 12_*_00*)*_ (σ) and *μ*’*_(H = 1_*_0000*)*_ (σ) for different Φ5 steel wires.

Specimen Number	Feature Parameters	Fitting Equation	R^2^
No.1	*μ*’*_(H = 12_*_00*)*_	*μ*’*_(H = 12_*_00*)*_ = −3.4345 × 10^−7^ *σ* + 8.3691 × 10^−4^	0.9890
	*μ*’*_(H = 1_*_0000*)*_	*μ*’*_(H = 1_*_0000*)*_ = 1.4798 × 10^−8^ *σ* + 4.3456 × 10^−6^	0.9948
No.2	*μ*’*_(H = 12_*_00*)*_	*μ*’*_(H = 12_*_00*)*_ = −3.4298 × 10^−7^ *σ* + 8.3679 × 10^−4^	0.9891
	*μ*’*_(H = 1_*_0000*)*_	*μ*’*_(H = 1_*_0000*)*_ = 1.4626 × 10^−8^ *σ* + 4.2536 × 10^−6^	0.9974
No.3	*μ*’*_(H = 12_*_00*)*_	*μ*’*_(H = 12_*_00*)*_ = −3.4626 × 10^−7^ *σ* + 8.4293 × 10^−4^	0.9892
	*μ*’*_(H = 1_*_0000*)*_	*μ*’*_(H = 1_*_0000*)*_ = 1.4773 × 10^−8^ *σ* + 4.2894 × 10^−6^	0.9970

**Table 4:** The fitting function and R-square of fitted curves *μ*’ *_(H = 12_*_00*)*_ (σ) and *μ*’ *_(H = 1_*_0000*)*_ (σ) for different Φ7 steel wires.

Specimen Number	Feature Parameters	Fitting Equation	R^2^
No.1	*μ*’*_(H = 12_*_00*)*_	*μ*’*_(H = 12_*_00*)*_ = −3.4404 × 10^−7^ *σ* + 8.325 × 10^−4^	0.9886
	*μ*’*_(H = 1_*_0000*)*_	*μ*’*_(H = 1_*_0000*)*_ = 1.4179 × 10^−8^ *σ* + 4.232 × 10^−6^	0.9902
No.2	*μ*’*_(H = 12_*_00*)*_	*μ*’*_(H = 12_*_00*)*_ = −3.446 × 10^−7^ *σ* + 8.2729 × 10^−4^	0.9879
	*μ*’*_(H = 1_*_0000*)*_	*μ*’*_(H = 1_*_0000*)*_ = 1.3432 × 10^−8^ *σ* + 4.4126 × 10^−6^	0.9896
No.3	*μ*’*_(H = 12_*_00*)*_	*μ*’*_(H = 12_*_00*)*_ = −3.4241 × 10^−7^ *σ* + 8.2994 × 10^−4^	0.9885
	*μ*’*_(H = 1_*_0000*)*_	*μ*’*_(H = 1_*_0000*)*_ = 1.468 × 10^−8^ *σ* + 3.7638 × 10^−6^	0.9904
